# The frequency of and risk factors for osteoporosis in Korean patients with rheumatoid arthritis

**DOI:** 10.1186/s12891-016-0952-8

**Published:** 2016-02-24

**Authors:** Joo-Hyun Lee, Yoon-Kyoung Sung, Chan-Bum Choi, Soo-Kyung Cho, So-Young Bang, Jung-Yoon Choe, Seung-Jae Hong, Jae-Bum Jun, Tae-Hwan Kim, Jisoo Lee, Hye-Soon Lee, Dae-Hyun Yoo, Bo Young Yoon, Sang-Cheol Bae

**Affiliations:** Department of Rheumatology, Inje University Ilsan Paik Hospital, Goyang, Republic of Korea; Department of Rheumatology, Hanyang University Hospital for Rheumatic Diseases, Seoul, 133-792 Republic of Korea; Department of Rheumatology, Hanyang University Guri Hospital, Guri, Republic of Korea; Department of Rheumatology, Catholic University of Daegu School of Medicine, Daegu, Republic of Korea; Department of Rheumatology, Kyung Hee University Hospital, Seoul, Republic of Korea; Department of Rheumatology, Ewha Womans University Mokdong Hospital, Seoul, Republic of Korea

**Keywords:** Osteoporosis, Rheumatoid arthritis, Frequency, Risk factors

## Abstract

**Background:**

The aim of this study was to investigate the prevalence of osteoporosis in rheumatoid arthritis (RA) patients and to analyze the risk factors in these patients using the KORean Observational study Network for Arthritis (KORONA) database.

**Methods:**

Among the RA patients in the KORONA who were recruited between July 2009 and December 2011, postmenopausal women with bone mineral density (BMD) results within one year from the time of KORONA enrollment were included in this study. The baseline characteristics of patients in three groups, defined by BMD results, were compared. The BMD measurement rates and prevalence of osteoporosis in the study patients were calculated in accordance with age and gender subgroups. Multivariable logistic regression analysis was used to explore the association between osteoporosis and demographics and disease-related risk factors.

**Results:**

Of 1322 postmenopausal woman patients with RA in whom BMD was measured within one year of study enrollment, 619 patients (46.8 %) were in the osteoporosis group (T-score ≤ −2.5 SD). RA patients with osteoporosis had a higher frequency of previous fractures than those in other groups, especially fractures of the femur (*p* = 0.004) and wrist (*p* = 0.042). Advanced age (≥70 years; OR = 2.28, 95 % CI: 1.40–3.58), lower body mass index (<25; OR = 2.14, 95 % CI:1.52–3.02), longer disease duration (≥10 years; OR = 1.46, 95 % CI: 1.07–2.00), higher cumulative glucocorticoid dose (OR = 1.03, 95 % CI: 1.01–1.05), and higher Health Assessment Questionnaire score (OR = 1.37, 95 % CI:1.11–1.69) were independent risk factors for osteoporosis.

**Conclusion:**

A large percentage (90.8 %) of RA patients enrolled in the KORONA cohort had osteoporosis and osteopenia. Nevertheless, BMD measurement rates in this population remained low, despite high risk groups of fractures.

## Background

Osteoporosis is a well-known extra-articular complication in patients with rheumatoid arthritis (RA) [1]. It is more common in patients with RA than in the general population, due to active systemic inflammation, the use of corticosteroids, and lack of mobility [2, 3]. As a result, patients with RA are at increased risk of fractures, an outcome that impairs quality of life and leads to mortality [4, 5].

The prevalence of osteoporosis in RA patients is reported to be approximately twice that in the general population [4]. The frequency of generalized osteoporosis in patients with RA ranges from 12.3 to 38.9 % in the lumbar spine and from 6.3 to 36.3 % in the hip [4–6]. Above all, there is at least a two-fold increase in the risk of vertebral fracture (VFs) in RA patients and a higher risk, up to six-fold, has been reported in patients with long-standing disease [6, 7]. However, studies on early RA have shown that VFs can be observed in the first year of the disease. As a result, approximately one-third of women with RA report a fracture within five years of follow up [8].

The risk factors for osteoporosis and osteoporotic fractures in RA patients are age, disability, low body mass index (BMI), previous non-vertebral fracture, long-standing disease, and glucocorticoids. In particular, regardless of additional risk factors, patients taking glucocorticoids should be screened for osteoporosis. Although awareness of osteoporosis by healthcare professionals has increased in recent years, it remains underdiagnosed and undertreated [9].

The increased risk of osteoporosis in RA patients is well reported [1, 2] and may be linked to differences in the distribution and interactions of genetic and environmental factors [10, 11]. However, little information is available on the frequency of osteoporosis and bone mineral density (BMD) measurement rates in RA patients and the associated risk factors in South Korea.

Thus, the aims of this study were to investigate the prevalence of osteoporosis in RA patients and to analyze the risk factors in these patients using the KORean Observational study Network for Arthritis (KORONA) database [12], a large, nationally representative Korean RA-specific cohort.

## Methods

### Study population

**Fig. 1:**
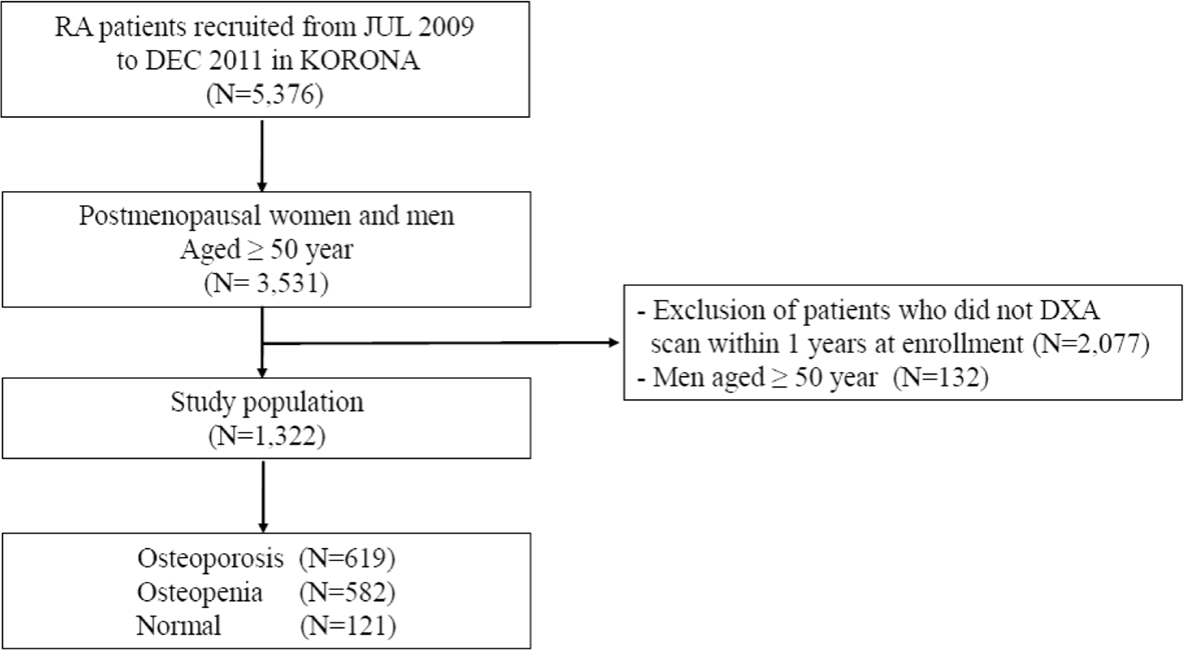
Flow diagram for the identification of study population in the KORONA. DXA, dual X-ray absorptiometry

KORONA was established in July 2009 by the Clinical Research Center for Rheumatoid Arthritis and funded by the Ministry of Health and Affairs, South Korea. RA patients over the age of 18 who satisfied the 1987 American College of Rheumatology (ACR) classification criteria for RA [13] were recruited by rheumatologists from 23 centers across South Korea as part of KORONA. Among those patients, only postmenopausal women were enrolled in this study. A total of 3531 patients with RA were recruited between July 2009 and December 2011, and 1322 postmenopausal women whose BMD examination results were available within one year from the time of KORONA enrollment were included in this study (Fig. 1). The KORONA protocol was approved by the institutional review boards of Hanyang University Hospital and all participating hospitals, and informed consent was obtained from all patients before registration.

### Data collection

#### Demographic and clinical features

All participants in this research completed an initial questionnaire to establish their demographic profile, medical history, and disease-specific outcomes. Disease activity was evaluated by physicians, and joint assessments were made by rheumatologists or well-trained health professionals. These evaluations, together with laboratory test results, prescription information (glucocorticoids—current and cumulative doses), and patient self-evaluation (history of previous fracture, fracture site, smoking, and alcohol intake), were incorporated into the KORONA database.

Laboratory data to identify the characteristics of RA (anti-cyclic-citrullinated protein antibody (ACPA) and rheumatoid factor (RF)) were obtained, and disease activity was monitored using erythrocyte sedimentation rate (ESR) and C-reactive protein (CRP). Disease Activity Score 28 using erythrocyte sedimentation rate (DAS28-ESR) and Health Assessment Questionnaire (HAQ) results were used to assess health-related outcomes. The DAS28-ESR index combines information regarding the number of swollen and tender joints, as well as a measure of general health and acute phase response (ESR) [14]. The HAQ is a model of patient-oriented outcome assessment and is based on the following five patient-centered dimensions: disability, pain, medication effect, cost of care, and mortality [15].

#### BMD data collection

Only patients with BMD data available within one year from the time of recruitment were included in this study. BMD measurements were carried out with Hologic QDR (Waltham, MA, USA) and GE Lunar Prodigy (Madison, WI, USA) systems, using the manufacturers’ standard scan and positioning protocols. Trained technicians at each center performed the scanning according to a standardized protocol. Each center followed the manufacturers’ procedures to check the stability and accuracy of their equipment using regular measurements of European phantom. However, no cross-calibration was performed on any of the systems. The reference BMD values use Korean data provided by the manufacturers.

Diagnoses of osteopenia and osteoporosis were made using calculations based on the World Health Organization (WHO) T-score criteria (−1.0 to −2.5 SD and ≤ −2.5 SD) [16]. In addition, according to the recommendation of the International Society for Clinical Densitometry, osteoporosis was diagnosed based on the lowest T-score among the three sites (lumbar spine, femoral neck, and total hip) [16].

### Statistical analysis

Baseline characteristics were compared among three patient groups (normal, osteopenia, and osteoporosis) defined according to BMD results (Table 1). Chi-square, Kruskal-Wallis test and one-way ANOVA were used for categorical, skewed continuous and continuous variables, respectively. BMD measurement rates within one year from the time of enrollment were analyzed, and the frequency of osteoporosis in the study patients was calculated for age subgroups (Table 2). Lumbar spine, femoral neck, and total hip T-scores in the enrolled RA patients were presented as the mean and standard deviation (SD) according to the patient’s age (Table 3). Finally, multivariable logistic regression analysis was used to explore the association between osteoporosis and the demographics and disease-related risk factors listed in Table 1 (Table 4). Some variables (HAQ, DAS28-ESR, ESR and CRP) that were not normally distributed were log-transformed to approximate a normal distribution for analysis. All analyses were performed using SPSS software (version 17.0, SPSS Inc., IL, USA). Results were considered statistically significant when p values were less than 0.05.

**Table 1:** Demographic and clinical features of study population

	Total	Osteoporosis	Osteopenia	Normal	*p*
	(*N* = 1322)	(*N* = 619)	(*N* = 582)	(*N* = 121)	
Age	61.2 ± 8.2	63.7 ± 8.2	59.8 ± 7.3	55.1 ± 6.6	<0.001
BMI (kg/m2)	22.9 ± 3.1	22.0 ± 3.2	23.4 ± 2.9	24.7 ± 3.3	<0.001
Disease duration (yrs)	12.1 ± 9.9	13.9 ± 10.6	10.9 ± −9.3	9.1 ± −8.7	<0.001
Previous fracture (*N*, %)					
Vertebrae	40 (3.0)	23 (1.7)	17 (1.3)	0 (0.0)	0.090
Femur	24 (1.8)	19 (1.4)	5 (0.4)	0 (0.0)	0.004
Wrist	66 (5.0)	38 (2.9)	27 (2.0)	1 (0.1)	0.042
Upper arm	16 (1.2)	11 (0.8)	5 (0.4)	0 (0.0)	0.154
Others^a^	171 (12.9)	95 (7.2)	65 (74.9)	11 (0.8)	0.041
Use of GC (*N*, %)	1027 (77.7)	498 (80.5)	439 (75.4)	90 (74.4)	0.074
Current dose of GC (mg/day)	4.3 ± 2.5	4.4 ± 2.7	4.2 ± 2.4	4.4 ± 2.1	0.581
Cumulative dose of GC (g*mo)^b^	5.4 ± 7.0	6.7 ± 7.7	4.4 ± 6.4	3.6 ± 6.1	<0.001
HAQ (0–3)	0.9 (1.0)	1.0 (1.1)	0.8 (0.9)	0.6 (0.9)	<0.001
DAS28-ESR	4.0 (1.8)	3.9 (1.9)	4.0 (1.8)	3.8 (1.5)	0.120
ESR (mm/h)	26.0 (32.0)	25.0 (32.0)	28.0 (33.0)	24.0 (27.0)	0.240
CRP (mg/dL)	0.3 (0.8)	0.3 (0.8)	0.3 (0.9)	0.2 (0.5)	0.058
RF, positivity (*N*, %)	1158 (87.7)	545 (88.2)	510 (87.6)	103 (85.1)	0.644
ACPA, positivity (*N*, %)	899 (84.3)	395 (83.5)	418 (85.5)	86 (81.9)	0.551

Values shown are median (interquartile range), mean ± standard deviation and frequency (percentage)

*BMI* bone mass index, *BMD* bone mineral density, *GC* glucocorticoid, *HAQ* health assessment questionnaire, *DAS 28-ESR* Disease Activity Score 28 using ESR, *ESR* erythrocyte sedimentation rate, *CRP* C-reactive protein, *RF* rheumatoid factor, *ACPA* anti-cyclic-citrullinated protein antibody

^a^Nasal bone, clavicle, shoulder, scapula, rib, hand, pelvic bone, ankle, feet, coccyx

^b^Dose of glucocorticoids according prednisolone × 30 × months/1000

**Table 2:** The frequency of osteoporosis at either the femoral neck or lumbar spine BMD with RA patients

Age	Total	BMD measurement rates	Osteoporosis	Osteopenia	Normal
	(*N*)	(*N*, %)	(*N*, %)	(*N*, %)	(*N*, %)
25–49	238	75 (5.7)	18 (2.9)	38 (6.5)	19 (15.7)
50–59	1267	503 (38.1)	174 (28.1)	257 (44.2)	72 (59.5)
60–69	1053	542 (41.0)	285 (46.0)	230 (39.5)	27 (22.3)
70–79	351	184 (13.9)	125 (20.2)	56 (9.6)	3 (2.5)
≥80	31	18 (1.4)	17 (2.8)	1 (0.2)	0 (0.0)
Total (*N*, %)	2940	1322 (45.0)	619 (46.8)	582 (44.0)	121 (9.2)

**Table 3:** T-scores in patients with RA according to age groups

Age	Lumbar spine^a^	Femoral neck	Total hip
	(*N* = 1317)	(*N* = 1277)	(*N* = 1266)
40–49	−0.9 ± 1.3	−1.2 ± 1.1	−0.6 ± 1.2
50–59	−1.6 ± 1.1	−1.6 ± 1.0	−1.1 ± 1.1
60–69	−1.9 ± 1.4	−2.1 ± 1.0	−1.6 ± 1.2
70–79	−2.1 ± 1.3	−2.5 ± 0.9	−2.1 ± 1.1
≥80	−2.9 ± 0.9	−2.6 ± 1.0	−2.7 ± 0.9
Total	−1.8 ± 1.3	−1.9 ± 1.1	−1.4 ± 1.2

^a^Average of T-score of L2-L4

**Table 4:** multivariale logistic regression analysis on risk factors of osteoporosis in RA patients

Risk factors	OR	95 % CI	*p*
Age			
< 70 yrs	1		
≥ 70 yrs	2.28	1.40–3.58	<0.001
BMI (Kg/m2)			
≥ 25	1		
< 25	2.14	1.52–3.02	<0.001
Disease duration (yrs)			
< 10 yrs	1		
≥ 10 yrs	1.46	1.07–2.00	0.032
Cumulative dose of GC (g)	1.03	1.01–1.05	0.009
RF, Positivity	0.86	0.55–1.36	0.690
ACPA, positivity	1.00	0.65–1.52	0.718
HAQ score	1.37	1.11–1.69	0.003
DAS28-ESR	0.69	0.39–1.24	0.217
ESR (mm/h)	0.94	0.75–1.18	0.571
CRP (mg/dL)	1.01	0.89–1.16	0.853

*OR* odds ratios, *CI* confidence interval, *BMI* bone mass index, *GC* glucocorticoid, *RF* rheumatoid factor, *ACPA* anti-cyclic-citrullinated protein, *HAQ* health assessment questionnaire, *DAS 28-ESR* Disease Activity Score 28 using ESR, *ESR* erythrocyte sedimentation rate, *CRP* C-reactive protein

## Results

### Demographic and clinical features

The demographic and clinical features of the RA patients are presented in Table 1. There were 1322 postmenopausal women with BMD results available within one year from the time of recruitment. The mean ages of these patients in the osteoporosis, osteopenia, and normal groups were 63.7 (8.2) years, 59.8 (7.3) years, and 55.1 (6.6) years, respectively; the patients with osteoporosis were significantly older than those with osteopenia or normal BMD (*p* < 0.001). Disease duration in the study population was 12.1 ± 9.9 years (mean). The RA patients with osteoporosis were older (*p* < 0.001) and had longer disease duration (*p* < 0.001), lower BMI (*p* < 0.001), and higher cumulative dosage of glucocorticoid (*p* < 0.001) than the patients in the other groups. The osteoporosis patients also had a higher frequency of previous fractures than the patients in the other groups, especially fractures of the femur (*p* = 0.004) and wrist (*p* = 0.042). HAQ scores (median and interquartile range) were significantly higher [1.0 (1.1)] in the osteoporosis patients than in the osteopenia patients [0.8 (0.9)] and the normal patients [0.6 (0.9)] (*p* < 0.001). Disease activity-related factors such as DAS28-ESR (*p* = 0.120), ESR (*p* = 0.240), CRP (*p* = 0.058), current smoking (*p* = 0.300), and alcohol intake (*p* = 0.067) did not differ among the three groups.

### Frequency of osteoporosis based on BMD

Among the 1322 patients with BMD data, 619 (46.8 %), 582 (44.0 %), and 121 (9.1 %) patients were in the osteoporosis, osteopenia, and normal groups, respectively. The number of osteoporosis patients in the 60–69 years age group was the highest (*n* = 285, 46.0 %) (Table 2). In addition, osteoporosis occurred in 31.2 % in the lumbar spine, 29.9 % in the femoral neck, and 18.5 % in total hip. The lumbar spine, femoral neck, and total hip T-scores in the entire cohort were analyzed according to each age subgroup. The femoral neck T-scores were lowest among the three sites in all age subgroups (Table 3).

### Risk factors for osteoporosis

The multivariable logistic regression modeling attempted to use all demographic and disease-related factors associated with the outcome in Table 1. As a result, advanced age (≥70 years; OR = 2.28, 95 % CI: 1.40–3.58), BMI < 25 (OR = 2.14, 95 % CI: 1.52–3.02), longer disease duration (≥10 years; OR = 1.46, 95 % CI: 1.07-2.00), higher cumulative dose of glucocorticoids (OR = 1.03, 95 % CI: 1.01–1.05), and higher HAQ score (OR = 1.37, 95 % CI: 1.11–1.69) were statistically significant independent factors associated with osteoporosis. However, disease activity-associated factors (ESR, CRP, and DAS28-ESR), RF and ACPA showed no independent association with osteoporosis (Table 4).

## Discussion

The aim of this study was to investigate the frequency of and risk factors for osteoporosis in Korean postmenopausal women with RA. The frequency of osteoporosis was 46.8 %. This frequency is higher in comparison to some other studies, which reported a prevalence of 22–24 % [17, 18]. However, a high prevalence (50 %) of osteoporosis was shown in a large Italian multicenter cross-sectional study that included 925 female RA patients [5]. In another study of postmenopausal women and men ≥50 years of age with seropositive RA in South Korea, 121 (52 %) of the 234 patients had osteoporosis [19]. This frequency was higher than that of the general population of females aged ≥50 years. In the Fourth Korea National Health and Nutrition Examination Survey database, it was estimated that in the general Korean female population aged ≥50 years, 39.1 % had osteoporosis while 43.4 % had osteopenia [20]. Thus, the prevalence rate of osteoporosis in RA patients varies among studies. This diversity can be explained by the use of different age groups in the study populations, the menopausal status of the participants, and the use of glucocorticoids.

BMD measurement is required in all RA patients due to the increased risk of fractures. However, a significant percentage of RA patients with fracture risk do not undergo BMD testing despite regular visits to rheumatologists. Of the 3531 RA patients enrolled in our study, only 41.2 % had BMD data. According to a study by the Consortium of Rheumatology Researchers of North America (CORRONA), of the RA patients in the database with at least a one-year follow up, 45 % reported dual-energy X-ray absorptiometry (DXA) data upon study entry. Furthermore, of the 2717 RA patients with at least a one-year follow up without reported DXA data at enrollment in the CORRONA study, 297 (11 %) patients reported undergoing DXA during the first year of follow up [21]. Although the CORRONA study included patients <50 years of age, the BMD measurement rate was higher than in this study. The patients in this study had a lower BMD measurement rate despite being older at enrollment. This finding should motivate Korean clinicians to carry out awareness and health education programs in these patients to improve the prevention, diagnosis, and treatment of osteoporosis.

Risk factors for developing osteoporosis in patients with RA include not only the use of glucocorticoids, high disability scores, low body weight, and age, but also RF status; the frequency of osteoporosis and reduced bone mass is higher in RF (+) patients. In our study, older age (≥70 years), low BMI (<25), longer disease duration (≥10 years), higher HAQ score, and higher cumulative glucocorticoid dose were significantly associated with osteoporosis. RF, ACPA, and the use of glucocorticoids were not significantly associated with osteoporosis (Table 1). Loss of BMD correlates with cumulative glucocorticoid dose, and a correlation has also been established between fracture risk and daily glucocorticoid dose [22]. A 30–50 % increase in fracture risk has been documented in patients receiving long-term glucocorticoid therapy in both cross-sectional and longitudinal studies [22, 23]. However, glucocorticoid therapy (used for controlling inflammation) had a positive effect on BMD in a placebo-controlled study conducted on RA patients [24, 25]. This effect could contribute to variability of BMD changes in response to glucocorticoid therapy. In our study, the use of glucocorticoids was not associated with osteoporosis; however, cumulative glucocorticoid dose was significantly associated with osteoporosis. Independent of RA, risk factors such as older age and low BMI are consistent with those in the general population [26]. However, disease activity (inflammation), lack of mobility, and treatment with corticosteroids, especially a high cumulative dose, are the main factors that increase the risk of osteoporotic fractures, on top of the background fracture risk based on advanced age, low BMI, and female gender, among others [27]. In our study, cigarette smoking and alcohol consumption were investigated as environmental factors; however, these factors were not found to be significantly associated with osteoporosis.

Fracture risk is highest among women with osteoporosis. However, many fractures occur in women with BMDs above the WHO threshold of −2.5 In a large cohort study, 82 % of women who sustained osteoporotic fractures of the wrist or forearm, hip, rib, or spine within one year after peripheral BMD testing had T-scores greater than −2.5 [28]. In the current study, the number of osteopenic patients (44.8 %) was comparable to that of osteoporotic patients (44.9 %). Nevertheless, no guidelines have yet been established for osteopenia patients in South Korea. Therefore, regular BMD screening and fracture risk assessment (such as Fracture Risk Assessment (FRAX®) is needed in RA patients, the high risk group for fractures [28].

To the best of our knowledge, this is the first study to investigate the prevalence of osteoporosis in RA patients using a large nationwide cohort (KORONA) in Korea. The numerous centers, both physician-derived and patient-derived outcomes, well controlled data, and use of a “real-world” cohort are the strengths of the study [12]. This study has a number of limitations. First, we cannot exclude the possibility of patient selection bias, because the 23 South Korean centers participating in this study were tertiary referral centers. The patients enrolled in this study had higher disease activity and functional disability than individuals not enrolled in this study. Therefore, BMD measurement rates in this study cannot represent the real rate of DXA in our country. Second, only patients with BMD data available within one year from the time of recruitment were included in this study. As a result, only about one-third of the population could be included in the analysis of risk factors for osteoporosis including BMD. Third, no phantom cross-calibration was performed among study centers. However, the centers were monitored with their local quality control phantoms and were found to be stable and calibrated to their manufacturer’s standards. Fourth, we used a cross-sectional study design; hence, it was not possible to make any cause–effect inferences about the relationship between RA characteristics and BMD. Therefore, further studies are needed to analyze the risk factors for osteoporotic fractures and anti-osteoporotic medication.

## Conclusions

We found that older age (≥70 years), low BMI (<25), longer disease duration (≥10 years), higher HAQ score, and higher cumulative glucocorticoid dose were significantly associated with osteoporosis. These findings were not markedly different from those of previous studies. A large percentage (90.8 %) of RA patients enrolled in the KORONA cohort had osteoporosis and osteopenia. BMD measurement rates in this population remain low, despite being a high-risk group for osteoporotic fractures.

## Abbreviation

BMD: bone mineral density
DXA: dual X-ray absorptiometry
KORONA: KORean Observational study Network for Arthritis
WHO: World Health Organization
